# Effects of Irbesartan and Amlodipine Besylate Tablets on the Intestinal Microflora of Rats With Hypertensive Renal Damage

**DOI:** 10.3389/fphar.2021.778072

**Published:** 2022-02-22

**Authors:** Jing Yu, Yan Ma, Xin He, Xiao Na Long, Jun Xu, Lei Wang, Zhi-Peng Feng, Hong-Ying Peng

**Affiliations:** ^1^ Department of Nephrology, The Affiliated Baiyun Hospital of Guizhou Medical University, Guizhou, China; ^2^ Department of Nephrology, The Affiliated Hospital of Guizhou Medical, Guizhou, China; ^3^ Department of Nephrology, The Bozhou County People’s Hospital, Guizhou, China

**Keywords:** irbesartan, amlodipine besylate tablets, hypertensive renal damage, urinary albumin, urea nitrogen, intestinal flora

## Abstract

**Objective:** The present study aimed to investigate the effects of irbesartan and amlodipine besylate tablets on the intestinal microflora of rats with hypertensive renal damage.

**Methods:** Eighteen 12-week-old male spontaneous hypertensive rats were randomly divided into three groups. The Ai-HDG group was given irbesartan at 15 mg/kg per day by gavage, the Ci-HDG group was given amlodipine besylate tablets at 1 mg/kg per day by gavage, and the Wi-HDG group, i.e., the control, was given the same dose of distilled water per day by gavage. The treatment lasted for 6 weeks. Six 12-week-old male Wistar–Kyoto rats were used as the reference group. Bacterial DNA was extracted from the feces of all the rats for high-throughput sequencing before and after the experiment. Operational taxonomic units were used to analyze the species of the intestinal flora, and the alpha diversity index was used to analyze the diversity. The relative abundance of the intestinal microflora in each group of rats was therefore analyzed at the phylum and genus levels.

**Results:** Compared with the Wi-HDG group, the alpha diversity of the Ai-HDG group increased (*p* < 0.05), while in the Ci-HDG group, only the Shannon index increased significantly. At the phylum level, compared with the control group, in the Ai-HDG and Ci-HDG groups, Firmicutes (F) decreased, Bacteroides (B) increased, and the F/B ratio decreased (*p* < 0.05). At the genus level, compared with the Wi-HDG group, the Ai-HDG and Ci-HDG groups did not show a significantly delayed decline in lactic acid bacteria. However, in the Ai-HDG group, the relative abundance of *Bifidobacteria* increased.

**Conclusion:** After the administration of irbesartan and amlodipine besylate, the disorder of intestinal flora in the rats with hypertensive renal damage improved. However, irbesartan was better than amlodipine besylate at improving the diversity of the intestinal flora in these rats.

## 1 Introduction

Hypertension is a disease that seriously affects human health. It is the most important risk factor for cardiovascular and cerebrovascular diseases and one of the three major causes of the global disease burden (GBD 2015 Risk Factors Collaborators, 2016). Hypertension can cause damage to the heart, kidneys, brain, and many other organs, placing a heavy burden on families and Communities. In China, aside from diabetic nephropathy and glomerulonephritis, hypertension is the most common cause of end-stage renal disease (17%), which is life-threatening. Hypertensive renal damage is damage to the renal structure and function caused by essential hypertension. Intestinal bacteria are known as the second gene pool of human beings and include over 400 species of bacteria with over 1,000 billion bacteria. These bacteria coexist and coordinate with each other to achieve balance and help maintain the health of our entire body, especially our intestines. It has been found that the existence and health of intestinal bacteria are closely related to a variety of diseases, such as cardiovascular disease, diabetes, and chronic kidney disease (Diamant et al., 2011; Pevsner-Fischer et al., 2016; Sampaio-Maia et al., 2016). Previous studies have revealed that intestinal flora plays an important role in the production of uremic toxins (Wikoff et al., 2009; Hsiao et al., 2013; Vanholder et al., 2014; Wong et al., 2014; Koppe et al., 2017; Mishima et al., 2017) and, thus, that probiotics could play a positive role in delaying the process of chronic renal failure (Vitetta et al., 2013; Sirich et al., 2014; Sonnenburg and Bäckhed, 2016; Chen and Vitetta, 2018; Thongprayoon et al., 2019). Irbesartan, an angiotensin ii receptor antagonist, can inhibit the activation of the AT1 pathway, which is induced by stress, thereby reducing the accumulation of reactive oxygen species and the inflammatory response in the intestines, restoring the expression of ACE2/B0AT1 and the activities of mTOR and P70S6K and inhibiting metabolic disorders and the tryptophan metabolism (Yisireyili et al., 2018). Several foreign scholars have used angiotensin II antagonists to study the brain–gut axis and believe that it can improve the intestinal flora, which could then be used for treating diseases. From November 2019 to January 2020, we observed changes in the intestinal flora of spontaneous hypertensive rats (SHR) following the intragastric administration of irbesartan and amlodipine besylate tablets. We analyzed the diversity of the intestinal flora to study the effects of these common antihypertensive drugs on hypertensive renal damage to provide a theoretical basis for the drugs’ clinical application.

## 2 Experimental Materials and Methods

### 2.1 Experimental Drugs, Reagents, Instruments, and Animal Feeding

This study was approved by the ethics committee of the Affiliated Baiyun Hospital of Guizhou Medical University. Eighteen 10-week-old male SHR and six 10-week-old male Wistar–Kyoto (WKY) rats were purchased from Beijing Weitong Lihua Experimental Animal Co., Ltd. Rats were fed for 2 weeks after purchase, and the adaptation time of the metabolic cage was 7 days. The rats were kept in individual ventitaled cages (IVC), and each group of rats were kept separately in a cage. Their drinking water was secondary purified water. Feeding environment: The temperature range was 20°C–25°C, and the relative humidity range was 40–70%. The rats’ blood pressure was measured weekly with a noninvasive blood pressure meter (Chengdu Taimeng, BP-300A). The SHR were randomly divided into three groups. The Ai-HDG group was given irbesartan (Shijiazhuang Yiling Pharmaceutical Co., Ltd., batch number A1908001) at 15 mg/kg per day by gavage, the Ci-HDG group was given amlodipine besylate tablets (Pfizer Pharmaceutical Co., Ltd., batch number AM2549, national drug approval no. H10950224) at 1 mg/kg per day by gavage, and the Wi-HDG group was the control group, and the same dose of distilled water was given by gavage every day. The continuous administration lasted 6 weeks.

### 2.2 Experimental Methods

Before administration, urine was collected from the metabolic cages of the rats in each group, and the samples were tested for urinary creatinine and microalbumin. The urinary creatinine of rats quantitative detection kit (enzyme linked immunosorbent assay) and the urinary microalbumin of rats quantitative detection kit (enzyme linked immunosorbent assay) were used to measure the urinary creatinine and microalbumin. The rats’ feces were collected under aseptic conditions and stored at −80°C. After administration, urine was collected once a week, and blood pressure was measured using a noninvasive blood pressure instrument once a week for 6 weeks. The blood pressure of rats was recorded after an average of 10 blood pressure measurements. In the process, the value deviation caused by technical problems was removed. The rats’ feces were again collected under aseptic conditions and stored at −80°C, and DNA was then extracted from the fecal samples and amplified by polymerase chain reaction (PCR). The final DNA concentration was determined by spectrophotometry, and the DNA quality was detected by 1% agarose gel electrophoresis. The V4 hypervariable region of the 16S rRNA gene was amplified by PCR, and the PCR products were further purified and sequenced by MiSeq (Illumina). The sequences of primers for PCR: 515F: GTGCCAGCMGCCGCGGTAA; 806R: GGACTACHVGGGTWTCTAAT. The intestinal microflora of the rats in each group was analyzed according to the phylum, genus, and biodiversity.

### 2.3 Statistical Analysis

The data were analyzed using R statistical software. The measurement data were expressed as the mean ± standard deviation (x ± SD) and compared using the rank sum test. *p* < 0.05 was considered statistically significant.

## 3 Results

**FIGURE1:**
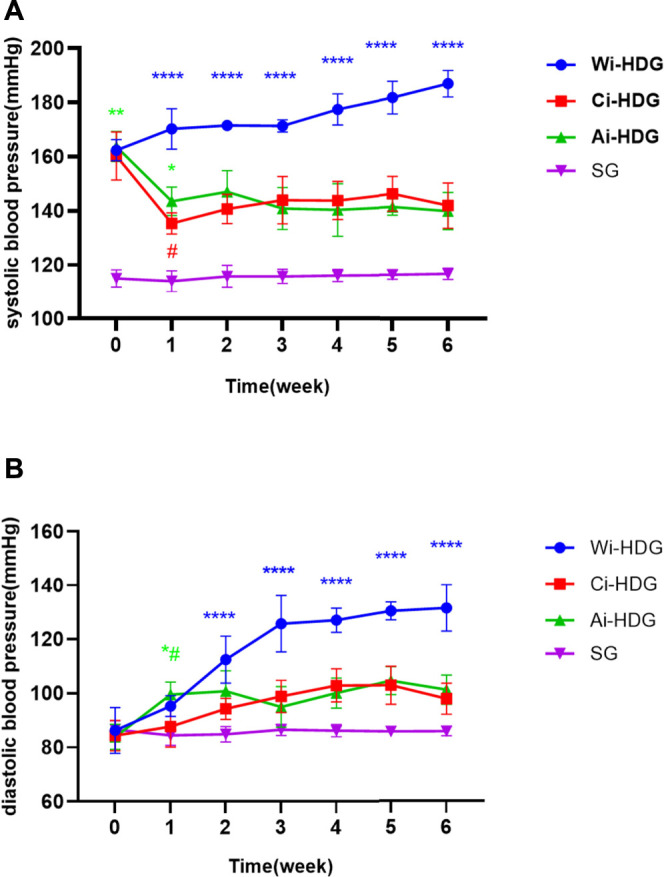
**(A)** Broken line chart of systolic blood pressure changes in rats in each group before and after gavage. **(B)** Broken line chart of diastolic blood pressure changes in rats in each group before and after gavage. The blood pressure was high before the SHR experiment. After administration, the blood pressure of rats in distilled water group continued to increase with the age of weeks. Comparing the changes of urinary creatinine and urinary micro protein in distilled water group before and after gavage, the diastolic and systolic blood pressure decreased after gavage in the irbesartan group and amlodipine besylate group. There were no significant changes in diastolic and systolic blood pressure between the irbesartan group and the amlodipine besylate group. Note: Compared with the distilled water group the change of before and after, ^#^ *p* < 0.05; and Compared with the SG group the change of before and after, *p < 0.05, **p < 0.01, ***p < 0.001, ****p < 0.0001.

**FIGURE 2:**
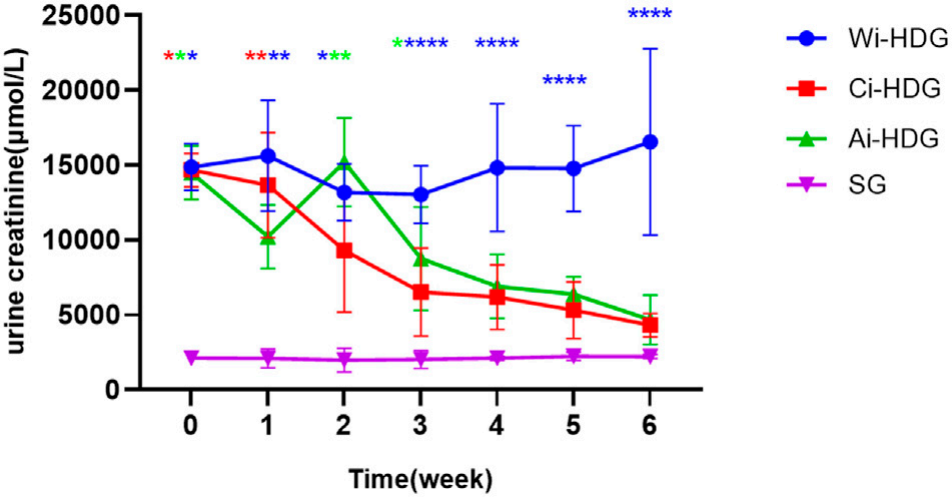
Broken line chart of urine creatinine changes in rats in each group before and after gavage.

**FIGURE 3:**
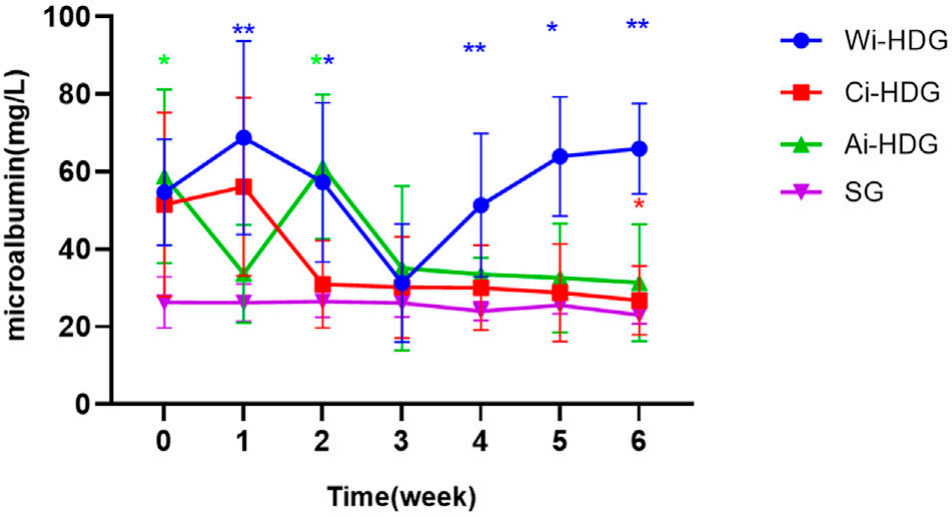
Broken line chart of microalbumin changes in rats in each group before and after gavage. The levels of urinary creatinine and urinary microalbumin collected by SHR before administration were significantly higher than those of WKY rats, which was consistent with the condition of hypertensive renal damage. Comparing the changes of urinary creatinine and urinary micro protein in distilled water group before and after gavage, amlodipine besylate group and irbesartan group decreased. There were no significant changes in urinary creatinine and urinary protein between the amlodipine besylate group and irbesartan group. Note: Compared with the distilled water group the change of before and after, ^#^ *p* < 0.05; and Compared with the SG group the change of before and after, *p < 0.05, **p < 0.01, ***p < 0.001, ****p < 0.0001.

A comparison of the rats’ blood pressure, urine creatinine, and microalbumin levels after gavage (x ± SD) is shown in Figures 1–3. Compared with the WKY rats, the levels of urinary creatinine (adjusted *p* < 0.001) and microalbumin (adjusted *p* = 0.007) increased in the hypertensive renal damage group, thereby demonstrating that the SHR used in this experiment were consistent with the condition of hypertensive renal damage. Before the experiment, the blood pressure in the SHR groups was high, and after administration, the blood pressure of the rats in the distilled water group continued to increase with age. Compared with the changes in the systolic blood pressure of the distilled water group, the systolic blood pressure of the irbesartan (adjusted *p* = 0.005) and amlodipine besylate (adjusted *p* = 0.021) groups decreased. Compared with the changes in the diastolic blood pressure of the distilled water group, the systolic blood pressure of the irbesartan (adjusted *p* = 0.021) and amlodipine besylate (adjusted *p* = 0.005) groups decreased after intragastric administration. There was no significant difference in the diastolic and systolic blood pressures between the irbesartan and amlodipine besylate groups. The urinary creatinine of the amlodipine besylate (adjusted *p* = 0.007) and irbesartan (adjusted *p* = 0.015) groups decreased. Compared with the distilled water group, the irbesartan (adjusted *p* = 0.024) and amlodipine besylate (adjusted *p* = 0.041) groups had a lower urinary microprotein level. There was no significant difference in urinary creatinine or protein between the amlodipine besylate and irbesartan groups. Therefore, it is clear that amlodipine besylate tablets and irbesartan can delay the development of hypertensive renal damage in rats.

**FIGURE 4:**
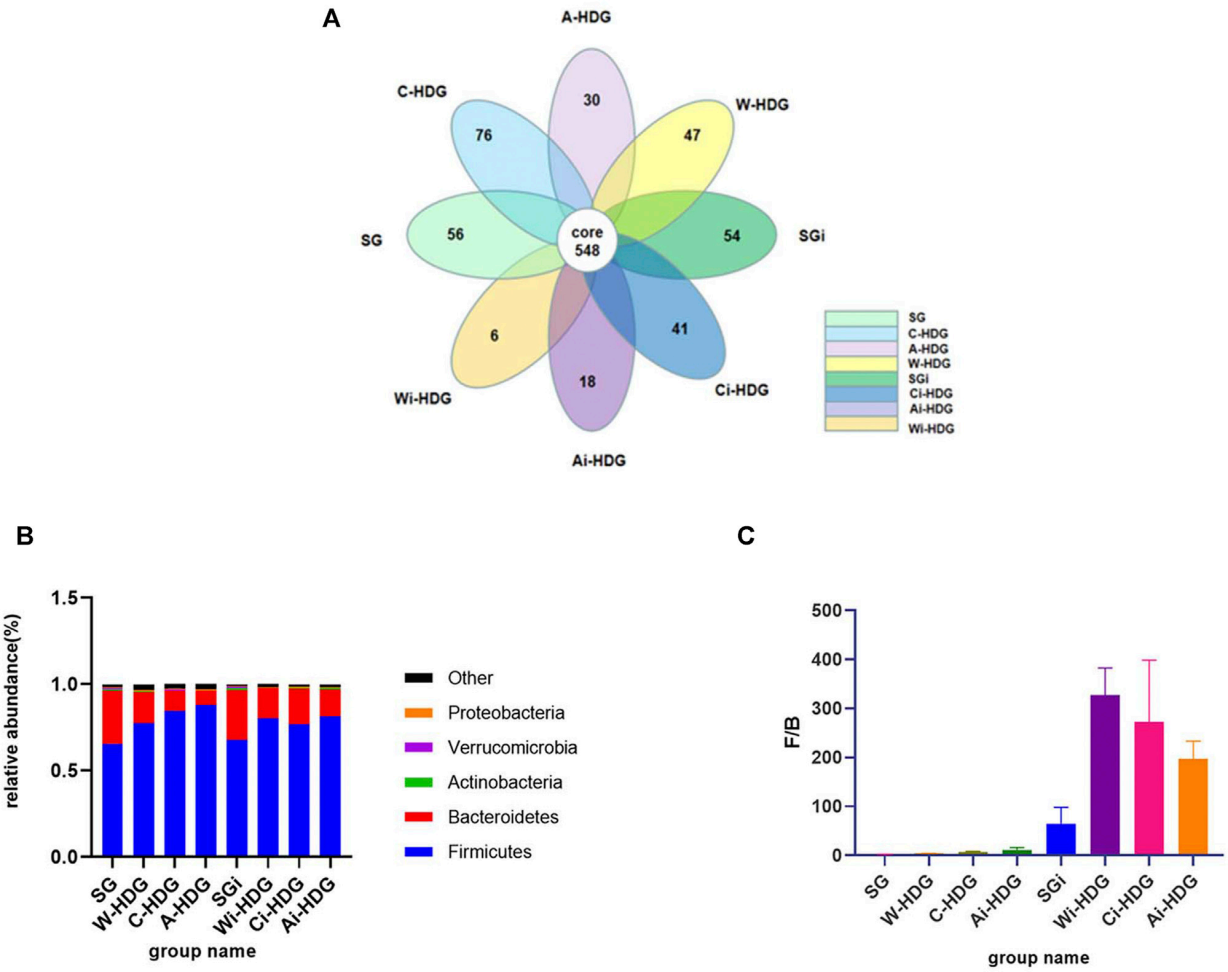
**(A)** The petal diagram of intestinal flora of rats in each group. With the progress of hypertensive renal damage in SHR, the specific flora of rats in each group decreased. **(B)** The column diagram of relative abundance of the intestinal flora of rats in each group at the portal level. **(C)** The column diagram of the F/B ratio in each group. At the gate level, compared with the distilled water group before and after gavage, the Firmicutes decreased, Bacteroidetes increased an F/B ratio decreased in the amlodipine besylate group and irbesartan group, indicating that the intestinal flora disorder and flora structure were improved in hypertensive renal damage rats after gavage of amlodipine besylate and irbesartan.

The observed species, Chao1 index, Simpson’s diversity index, Shannon index, and phylogenetic diversity (PD_whole_tree) of the rats in each group (x ± SD mg/L) after administration are shown in Table 1. The alpha diversity analysis reflects the abundance and diversity of the flora within each group, while the Chao1, observed species, PD_whole_tree, Shannon, Simpson’s diversity, and Good’s coverage indexes reflect the diversity of the flora within a group. Good’s coverage reflects the sequencing depth of the samples, which was 99.0% in each group, indicating that the sequencing depth was sufficient. The alpha diversity analysis indexes (Shannon, Simpson’s diversity, Chao1, and PD_whole_tree) of the different samples under a 97% consistency threshold were compared according to the comparison of the rats in each group before and after gavage. The observed species (H = 9.589, *p* = 0.008) and Shannon index (H = 11.415, *p* = 0.003) changes in the rats with hypertensive renal damage in the distilled water, amlodipine besylate, and irbesartan groups before and after intragastric administration as well as the Simpson’s diversity index (H = 10.716, *p* = 0.005), Chao1 index (H = 11.415, *p* = 0.003), and PD_whole_tree index (H = 11.789, *p* = 0.003) changes had statistically significant differences. A pairwise comparison showed changes in observed species between the rats in the distilled water and irbesartan groups (adjusted *p* = 0.017), changes in the Shannon index (adjusted *p* = 0.007), and changes in the Simpson’s diversity index (adjusted *p* = 0.007). The difference between the Chao1 index (adjusted *p* = 0.007) and the PD_whole_tree index (adjusted *p* = 0.004) was statistically significant. Regarding the pairwise comparison of the distilled water and amlodipine besylate groups, the Shannon index (adjusted *p* = 0.015) difference was statistically significant, while the observed species, Chao1 index, Simpson’s diversity index, and PD_whole_tree changes were not statistically different. Moreover, regarding the pairwise comparison of the observed species (adjusted *p* = 0.028), Simpson’s diversity index (adjusted *p* = 0.033), and Chao1 index (adjusted) changes between the irbesartan and amlodipine besylate groups (adjusted *p* = 0.015) and the PD_whole_tree index changes (adjusted *p* = 0.028), the difference was statistically significant; however, the Shannon index changes were not. It was found that the improvement in the diversity of the microbial communities in the Ai-HDG group may have been more significant than that of the other groups. The petals of the common and unique operational taxonomic units among the different sample groups are shown in Figure 4A. The relative abundance of the intestinal flora in each group at the phylum level is shown in Figure 4B. The F/B ratio in each group is shown in Figure 4C.

**TABLE 1:** Observed species, Chao1 index, Simpson’s diversity index, Shannon index and PD_whole_tree X ± s (mg/L) of rats in each group before and after administration of medicine.

Constituencies		Observed species	Chao 1 index	Simpson’s diversity index	Shannon index	PD_whole_tree
Before intervention	W-HDG group	669.83 ± 43.86	739.69 ± 93.14	0.88 ± 0.09	5.83 ± 0.78	37.11 ± 5.20
	C-HDG group	690.33 ± 42.68	741.68 ± 146.27	0.87 ± 0.06	4.71 ± 1.11	33.92 ± 5.74
	A-HDG group	600.83 ± 38.88	652.03 ± 40.41	0.78 ± 0.06	4.69 ± 0.83	36.26 ± 5.85
After intervention	Wi-HDG group	753.83 ± 17.01	801.60 ± 20.44	0.94 ± 0.05	6.37 ± 0.49	37.63 ± 2.15
	Ci-HDG group	794.6 ± 41.38	809.96 ± 97.49	0.96 ± 0.01	6.92 ± 0.36*	35.92 ± 2.98
	Ai-HDG group	761.17 ± 20.06*^#^	813.83 ± 21.56^*#^	0.97 ± 0.01*^#^	6.76 ± 0.38*	42.46 ± 5.62*^#^

Note: Compared with the distilled water group the change of before and after, *p < 0.05; and Compared with the amlodipine besylate tablet group the change of before and after, ^#^ *p* < 0.05.

Comparison at the phylum level: Most of the bacteria in the intestines were Firmicutes, Bacteroides, Actinomycetes, Proteus, and Verrucomicrobia. There were significant differences in the relative abundance of Firmicutes (H = 11.661, *p* = 0.003) and Bacteroides (H = 11.789, *p* = 0.003) as well as in the changes in the Firmicutes/Bacteroides (F/B) ratio (H = 11.942, *p* = 0.003) of the three SHR groups before and after intragastric administration. The changes in the relative abundance of the Firmicutes (adjusted *p* = 0.004) and Bacteroides (adjusted *p* = 0.028) and the changes in the F/B ratio (adjusted *p* = 0.033) were significantly different between the distilled water and amlodipine besylate groups. There were also significant differences in the changes in the abundance of Firmicutes (*p* = 0.024), Bacteroides (*p* = 0.004), and the F/B ratio (*p* = 0.003) between the distilled water and irbesartan groups. However, there was no significant difference in the changes in the relative abundance of the Firmicutes or Bacteroides and the changes in the F/B ratio between the irbesartan and amlodipine besylate groups (Figures 44B,C).

Comparison of the relative abundance of probiotics (*Bifidobacterium*, *Lactobacillus*) at the genus level: There was no significant difference in the change in the relative abundance of the lactic acid bacteria before and after the intragastric administration of distilled water, amlodipine besylate, and irbesartan. The change in the relative abundance of *Bifidobacteria* in the groups before and after intragastric administration (H = 10.239, *p* = 0.006) was statistically significant. The difference between the distilled water and amlodipine besylate groups (adjusted *p* = 0.026) was statistically significant, but there was no significant difference between the distilled water and irbesartan groups (Table 2).

**TABLE 2:** The relative abundance ratio of Bifidobacteria and Lactobacillus in each group before and after administration of medicine $\bar{\text{X}}$ ± s (‰).

Constituencies	W-HDG group	C-HDG group	A-HDG group	WI-HDG group	CI-HDG group	AI-HDG group
Bifidobacteria	0.09 ± 0.05	0.12 ± 0.10	0.03 ± 0.03	0.07 ± 0.05^#^	0.23 ± 0.16*	0.01 ± 0.01^#^
Lactobacillus	476.32 ± 100.96	497.15 ± 61.52	467.80 ± 97.02	187.79 ± 26.18	186.60 ± 56.86	232.03 ± 91.22

Note: Compared with the distilled water group the change of before and after, *p < 0.05, and Compared with the amlodipine besylate tablet group the change of before and after, ^#^ *p* < 0.05.

## 4 Discussion

Healthy intestinal flora can protect the body against the invasion of harmful microorganisms and ensure the integrity of the intestinal mucosa. The flora affects the body’s immunity, insulin sensitivity, weight, and even the functioning of the cognitive part of the brain (Fukuda et al., 2011; Blumberg and Powrie, 2012; Nicholson et al., 2012; Luck et al., 2015; Rosenbaum et al., 2015). The flora may also play an important role in the occurrence and development of disease. In the colon, anaerobic symbiotic microorganisms can partially ferment digestible and non-digestible carbohydrates (dietary fiber) to produce short-chain fatty acids (SCFAs), which can be used as an energy source for epithelial cells, maintain the epithelial barrier, protect mucosal immunity, and affect the biological functioning of regulatory T cells (Tregs). The acetate in SCFAs can enhance their anti-infection function to maintain the integrity of intestinal epithelial cells. Moreover, the butyrate in SCFAs can cause goblet cells to upregulate mucin gene expression to help protect mucosal immunity, enhance the forkhead/winged helix transcription factor p3 gene promoter, and retain acetylation of histone H3 with a non-coding sequence, inducing Treg differentiation to improve colitis (Arpaia et al., 2015). A previous study revealed that the consumption of butyrate-producing bacteria may be related to chronic kidney disease (CKD)-related inflammation and processes (Jiang et al., 2016). Uremic toxins that cannot be removed by dialysis, particularly the serum levels of indoxyl sulfate (IS) and p-cresol sulfate, are closely related to the progress of CKD. IS is completely derived from the intestinal fermentation of dietary substances; bacterial tryptophanase converts tryptophan into indole, which is then absorbed and processed by the host to produce IS. The manipulation of tryptophanase from Bacteroides can reduce the amount of indole produced, which in turn reduces the circulating IS level. This suggests that microbiota could be used in the treatment of nephropathy (Devlin et al., 2016). It has previously been proven that *Prevotella* can produce SCFAs, which can reduce inflammation in acute kidney injury (Andrade-Oliveira et al., 2015).

On detecting the 16S RNA of the fecal flora in hypertensive rats and patients, Jama et al. (2019) revealed that the abundance and diversity of the intestinal flora decreased significantly and that the F/B ratio increased. *Proteus*, which is more abundant in patients with diabetic nephropathy, is also effective at increasing the circulating lipopolysaccharide levels (Rizzatti et al., 2017; Wassenaar and Zimmermann, 2018). Chronic kidney disease is usually accompanied by a decline in the diversity of the host intestinal flora and flora disorder, which can further aggravate renal function damage. In this study, compared with the WKY rats, the rats in the Wi-HDG group had more Firmicutes, less Bacteroides, and a higher F/B ratio, indicating that there was a disorder in the intestinal flora of the hypertensive rats with renal damage.

Diet and drugs achieve therapeutic effects by affecting the intestinal flora. In a population that eats more protein and animal fat, Bacteroides dominate in the intestines; in a population whose food is mostly comprised of carbohydrates, *Prevotella* dominate in the intestines (Wu et al., 2011). The causal relationship between renal function damage and intestinal flora, the exact mechanism, and the development of related research in relation to the human body still need to be further explored. The relationship between intestinal microflora and renal dysfunction is mainly through malnutrition, SCFAs, the gut renal axis, the renin–angiotensin system, the inflammation and immune response, and uremic toxins. In this experiment, compared with the Wi-HDG group, the urine creatinine and urinary albumin decreased in the Ai-HDG and Ci-HDG groups, demonstrating that the drugs used in this study could protect the renal function of rats with hypertensive renal damage. Compared with the changes in the diversity index before and after gavage in the distilled water group, diversity in the irbesartan group increased. Therefore, as the species richness increases, this probability decreases. While the study results suggest that irbesartan increases the diversity of the intestinal flora in the SHR groups, after the intervention with amlodipine besylate tablets, only the Shannon index increased significantly. There was no significant change in the other related indexes compared with the distilled water intervention group. The Shannon index represents the disorder and uncertainty of the individual species, the higher the uncertainty, the higher the diversity. The Shannon index contains two factors: 1) The number of species in terms of richness, 2) The uniformity of the individual distribution of a species. Therefore, it could be concluded that the species distribution of the intestinal flora in the amlodipine besylate group was uniform; there was no indication of increased species diversity. Compared with the changes in the distilled water group, the analysis at the phylum level revealed that in the Ai-HDG and Ci-HDG groups, Firmicutes decreased, Bacteroides increased, and the F/B ratio decreased, demonstrating that the intestinal microflora structural disorder in the rats with hypertensive renal damage was improved. The comparison of the relative abundance of the probiotics *Bifidobacterium* and *Lactobacillus* at the genus level revealed that compared with the Wi-HDG group, the Ai-HDG and Ci-HDG groups did not show a significant delay in the decline of lactic acid bacteria. However, in the Ai-HDG group, the relative abundance of *Bifidobacteria* increased significantly. In conclusion, after the administration of irbesartan and amlodipine besylate, the structural disorder of the intestinal flora in the rats with hypertensive renal damage improved. Irbesartan was better than the amlodipine besylate tablets at improving the diversity of the intestinal flora in these rats. After the administration of irbesartan and amlodipine besylate, the intestinal flora and the renal function in these rats improved; however, it is still not clear whether the two drugs can improve the intestinal flora, which improves renal function, or whether they improve renal function directly. Further experiments are needed to prove the causality.

## 5 Conclusion

In recent years, there has been an increasing amount of research on the relationship between intestinal flora and disease. Intestinal flora may become an appropriate therapeutic target in the future, but in the treatment of chronic disease, the intestinal flora of patients can change. This study not only provides a basis for the application of the common antihypertensive drugs amlodipine besylate and irbesartan in the treatment of hypertensive renal damage but also provides novel information that enables patients with chronic disease to choose the appropriate flora-targeted treatment when their intestinal flora change after the oral administration of drugs. This may be a future direction for the treatment of disease.

## Data Availability

The original contributions presented in the study are included in the article/supplementary material, further inquiries can be directed to the corresponding author. The data presented in the study are deposited in the NCBI repository, accession number: PRJNA792899.
